# A dynamic variant of Takotsubo cardiomyopathy mimicking apical hypertrophic cardiomyopathy: a case report

**DOI:** 10.1093/ehjcr/ytae432

**Published:** 2024-08-23

**Authors:** Chitsa Seyani, Robert Adam, David Gant, James Shambrook, Andrew Flett

**Affiliations:** Cardiovascular and Thoracic Division, Cardiology Department, University Hospital Southampton, Tremona Road, Southampton SO16 6YD, UK; Cardiovascular and Thoracic Division, Cardiology Department, University Hospital Southampton, Tremona Road, Southampton SO16 6YD, UK; Cardiovascular and Thoracic Division, Cardiology Department, University Hospital Southampton, Tremona Road, Southampton SO16 6YD, UK; Cardiovascular and Thoracic Division, Cardiology Department, University Hospital Southampton, Tremona Road, Southampton SO16 6YD, UK; Cardiovascular and Thoracic Division, Cardiology Department, University Hospital Southampton, Tremona Road, Southampton SO16 6YD, UK

**Keywords:** Transient apical wall thickening, Takotsubo cardiomyopathy, Apical ballooning Syndrome, Case report, Cardiac magnetic resonance Imaging, Parametric mapping, Apical hypertrophic cardiomyopathy

## Abstract

**Background:**

Takotsubo cardiomyopathy usually presents with acute reversible left ventricular apical hypokinesia and apical ballooning with basal hyperdynamic function. We describe an underreported case of Takotsubo cardiomyopathy (TCM), misinterpreted as apical hypertrophic cardiomyopathy (HCM) due to transient apical oedema in the recovery phase of the condition.

**Case summary:**

A 74-year-old Caucasian woman, presented to the emergency department complaining of retrosternal chest pain following, emotional stress. Her electrocardiogram (ECG) demonstrated poor R-wave progression in the right precordial leads raising the possibility of an anterior non-ST elevation myocardial infarction. High sensitivity troponin was elevated at 2232 ng/L, echocardiography demonstrating apical ballooning and left ventricular ejection fraction of 45%. Computed tomography coronary angiography revealed unobstructive coronary arteries. Cardiac magnetic resonance imaging (cMRI) demonstrated morphological features consistent with apical HCM. However, subsequent cMRI examination, illustrated resolution of the hypertrophic segments, with T1 parametric mapping and extracellular volume (ECV) favouring TCM over apical HCM.

**Discussion:**

Following emotional stress, takotsubo cardiomyopathy is understood caused by various mechanisms: catecholamine surge, inflammation, or transient ischaemia event leading to dysregulated cellular mechanisms. This results in myocardial oedema and increased wall stiffness leading to non-type 1 coronary plaque event-related regional wall motion abnormalities.

In this instance, the initial two cMRI studies reported morphological features consistent with apical HCM, however, a subsequent examination demonstrated resolution of these features. The key to delineating these distinct pathologies lies in myocardial T1 times that exceeded what is normally observed in HCM, the absence of late gadolinium enhancement and ECV values which favour takotsubo cardiomyopathy.

**Learning points**

- Takotsubo is a stress cardiomyopathy leading to extreme changes in patient physiology, correct differential diagnosis from acute coronary syndrome is fundamental as subjecting individuals to invasive coronary percutaneous coronary intervention is not without its risks.
- Clinicians must be aware that in the recovery phase from takotsubo cardiomyopathy, the left ventricle undergoes morphological changes that may mimic apical hypertrophic cardiomyopathy and a systematic approach to cardiac MRI interpretation including parametric mapping is key to differentiating these two diagnoses.

## Introduction

Takotsubo cardiomyopathy (TCM) is increasingly recognized inpatients who present with troponin-positive chest pain and unobstructed coronary arteries. Although pathophysiology is not well understood, hypotheses include a catecholamine surge, inflammation, or transient ischaemia. Echocardiography illustrates hyperkinetic basal segments with hypokinesis of the mid-septum to apical walls.^1^

Apical hypertrophic cardiomyopathy (HCM) is a variant in the spectrum of HCM. It is most frequently observed in Asian communities with a prevalence up to 25%, compared to 1% – 10% in individuals of European heritage.^2^

In this case report, we present a Caucasian woman with TCM, who then developed apical myocardial oedema (pseudohypertrophy) mimicking apical HCM in the recovery phase. Our aim in this report is to: (i) highlight this interesting clinical scenario, (ii) stress caution in making a diagnosis of apical HCM in this clinical context, and (iii) demonstrate the role of cardiac magnetic resonance imaging (cMRI) as a diagnostic tool.

## Summary figure

**Figure:**
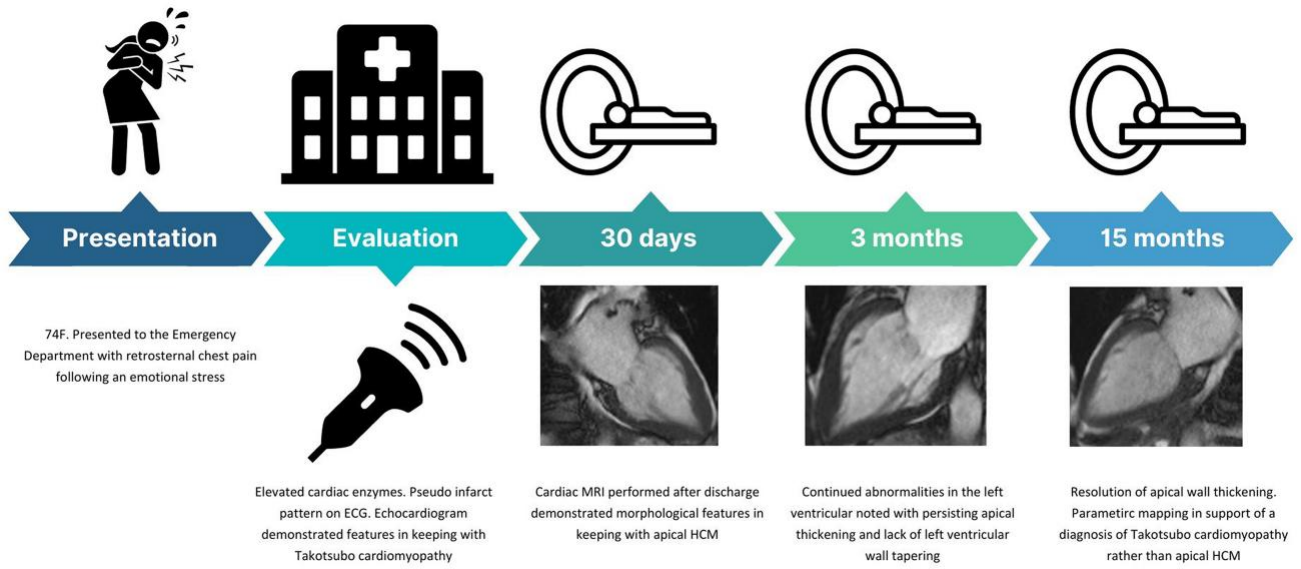


## Case presentation

A 74-year-old Caucasian woman presented to the emergency department complaining of retrosternal chest pain following emotional stress, her symptoms resolved spontaneously on arrival. Initial observations were unremarkable: oxygen saturations 97% on room air, blood pressure 105/57 mmHg, respiratory rate 17 breaths/min, heart rate 75 beats/min, and she was apyrexial. Clinical examination revealed a very cachectic woman, jugular venous pressure was not elevated, cardiopulmonary auscultation was normal. There were no peripheral signs of cardiac decompensation.

Initial troponin was elevated, 2008 ng/L and peaked at 2232 ng/L (reference range < 18 ng/L). Bedside echocardiography demonstrated apical ballooning consistent with TCM with a left ventricular ejection fraction of 45%.

Past medical history included systemic sclerosis, hypoparathyroidism, hereditary haemorrhagic telangiectasia, low body mass index of 14, colon cancer resected 39 years ago resulting in short bowel syndrome. She had historic management of paroxysmal supraventricular tachycardia by cardiovascular services. Regular medications included flecainide 50 mg, prednisolone 12.5 mg, omeprazole 40 mg, and replacement therapies for hypoparathyroidism.

**Figure 1:**
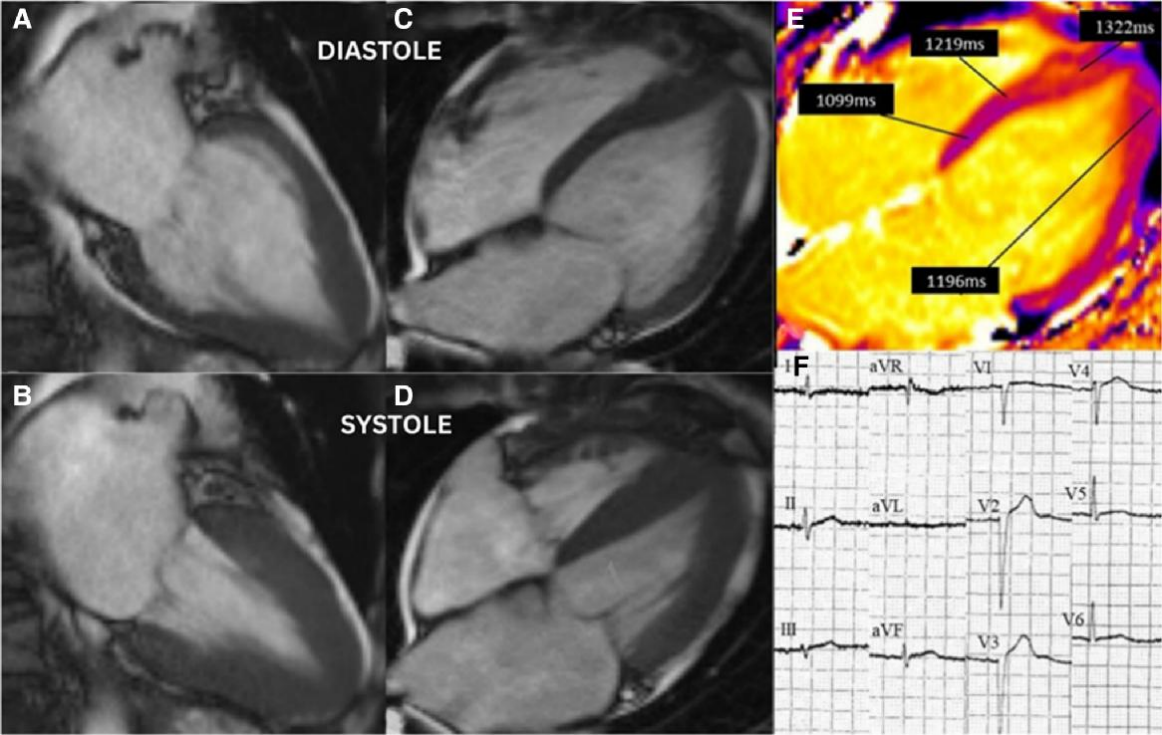
Baseline cardiac MRI. Diastolic, (*A*, *C*) and systolic phases, (*B*, *D*). Lack of basal to apical wall tapering, (*C*). Elevated mid to apical T1 mapping times (*E*). Admission ECG with poor R-wave progression, (*F*).

In view of her electrocardiogram (ECG) raising concerns of an anterior non-ST elevation myocardial infarct (NSTEMI) (*Figure 1F*), together with elevated cardiac troponins, she was initially diagnosed as having an acute coronary syndrome (ACS). Initial management consisted of single antiplatelet therapy, clopidogrel 75 mg once daily, as she could not tolerate aspirin due to a history of bleeding.

The patient was reviewed by the ACS team, the history of emotional stress prior to the development of pain, echocardiographic findings suggestive of apical ballooning, patient frailty, and multimorbid state, computed tomography coronary angiography (CTCA) was undertaken in the first instance which demonstrated minimal ostial disease of the left main stem, mild stenosis of the left anterior descending artery, mild stenosis of circumflex artery, and moderate stenosis of the right coronary. A multidisciplinary decision, involving the patient and the ACS team, was made to manage her conservatively due to her multimorbid state, and intolerance of aspirin. After a period of observation, she was discharged on clopidogrel 75 mg, ramipril 1.25 mg, atorvastatin 80 mg, and flecainide was stopped in favour of nebivolol 1.25 mg.

A non-stress cMRI was arranged a month later to elucidate the diagnosis of ACS or TCM. This demonstrated an improving ejection fraction of 59%. Incidentally, the cMRI showed morphological features consistent with apical HCM (*Figure 1*). Maximum wall thickness was 15 mm (normal range < 12 mm) in the apical segments compared to 7 mm in the basal segments. T1 times were elevated at 1219 ms (local normal T1 reference < 1060 ms) in the mid-septum, rising to 1322 ms in the apical septum. T1 relaxation times in the apex were interpreted as diffuse fibrosis, although no focal scarring was observed on late gadolinium enhancement (LGE). There was no evidence of myocardial infarction. Antiplatelet therapy was stopped with recommendations to continue with beta blockade and angiotensin converting enzyme inhibition therapy.

**Figure 2:**
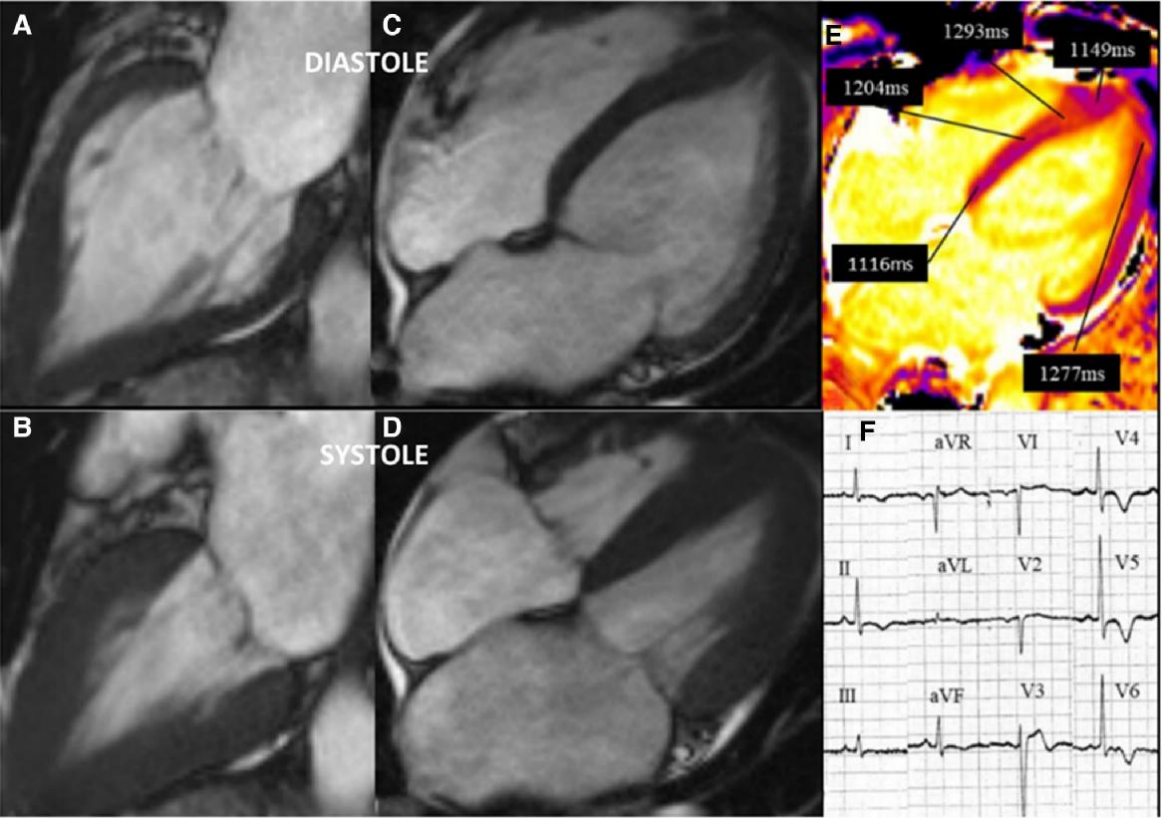
Cardiac MRI at 3 months. Resolution of the apical wall thickening, (*A*, *C*) diastole, (*B*. *D*) systole. T1 mapping times show ongoing elevation of T1times in the apex compared to the base, (*E*). Evolving ECG changes mimicking apical hypertrophic cardiomyopathy criteria, (*F*).

**Figure 3:**
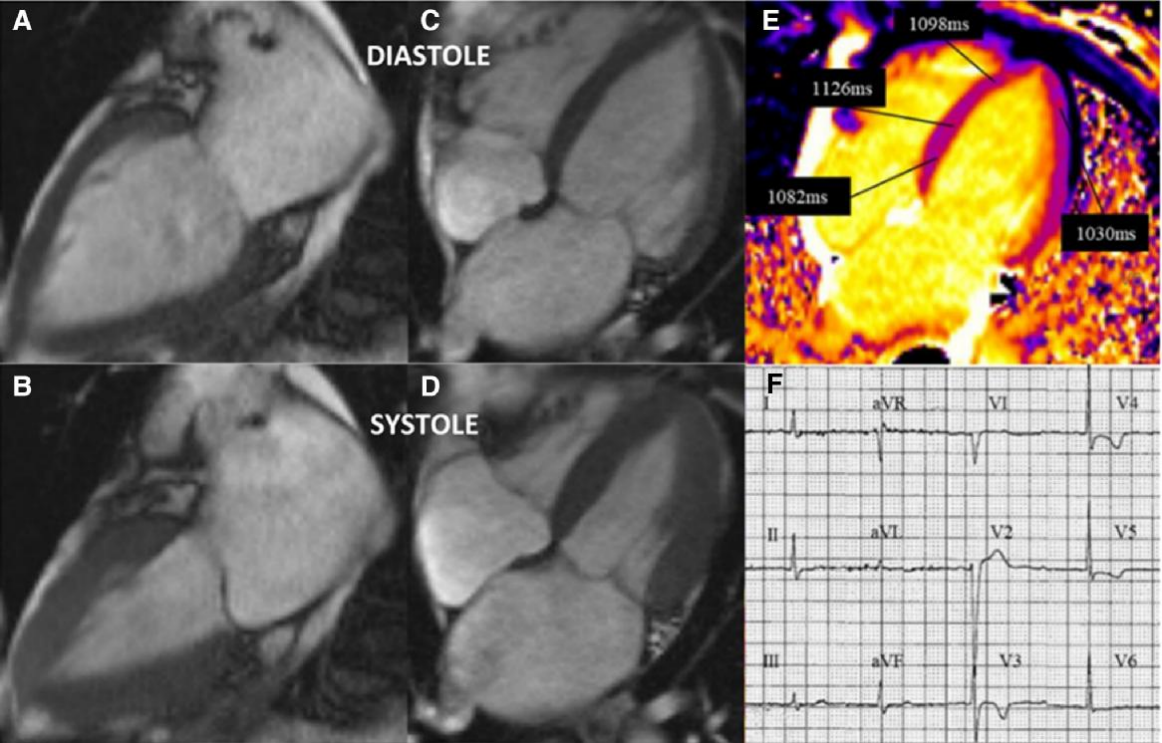
Cardiac MRI at 15 months. Complete resolution of apical wall thickening, (*A*, *C*) diastole, (*B*, *D*) systole. Resolving T1 times, (*E*) and persisting T-waves changes (*F*).

The patient represented 3 months after discharge with supraventricular tachycardia, chest pains, and elevated cardiac enzymes. Subsequent cMRI (*Figure 2*), did not demonstrate any focal LGE, but persistently elevated T1 times 1204 ms in the mid-septum and 1293 ms in the apical septal wall. Minor reduction of increased wall thickness of the apical segments was noted with improved contractility compared with previous examination. Her clinical presentation was thought to be in part due to a known history paroxysmal supraventricular tachycardia in the recovery phase of TCM. She was discharged with an increased dose of nebivolol with a planned outpatient clinic review. Due to the discrepancy between the two initial cMRI investigations, a further cMRI examination was requested, which took place 15 months after her index presentation (*Figure 3*). Her cMRI revealed complete resolution of the apical hypertrophy with near normalization of T1 times of 1082 ms in the basal-septum and 1098 ms in the apical septum. No LGE was noted at this time, and the patient reported complete resolution of symptoms.

## Discussion

Apical HCM exists within the spectrum of HCM, it is commonly observed in individuals of Asian heritage and less so in the European and North American individuals.^3^ Myosin binding protein C (MYBPC3) and beta-myosin heavy chain (MYH7) gene mutations account for 50% of this phenotype. Diagnostic criteria include changes in the apex of the ventricle, often described as a spade-like morphology, precordial T-wave inversion on ECG, apical wall thickness of > 15 mm, lack of apical wall tapering or base to apical wall ratio > 1.5.^4^ In contrast, TCM presents as an acute reversible cardiomyopathy with hyperdynamic basal segments and apical hypokinesia. The patient is often a post-menopausal woman presenting with acute chest pain, elevated biomarkers of myocardial injury and ECG changes due to emotional stress.^5^ Coronary imaging frequently reveals unobstructed coronary arteries and echocardiographic features of apical ballooning.

Essential elements to highlight in relation to our case favouring TCM over apical HCM were the antecedent stressor in her clinical presentation, low prevalence of apical HCM in the Caucasian ethnic group and being post-menopausal. It was appropriate to consider TCM as well as ACS pending further investigations. CT coronary angiography demonstrated unobstructive coronary artery disease, resulting in optimization of heart failure therapy.

Earlier reports describe similar cases of patients presenting with TCM, unobstructed coronary arteries and echocardiographic findings that demonstrated reduced left ventricular ejection fraction.^6,7^ Evolutionary changes of transient apical wall thickening were historically described by echocardiography before the prevalence of cMRI increased.

The added value of cMRI in the diagnostic pathway is well established in the literature with a case of a post-menopausal woman presenting with recurrent episodes of TCM, with similar morphological features to this reported case observed on cMRI including resolution over 6 months, suggesting this phenomenon can be recurrent.^8^

During the acute phase of TCM, there is increased myocardial oedema and efflux of collagen into the extracellular matrix, it is this process that makes cMRI a valuable diagnostic tool.^1,9^ This pathophysiological process is visualized on cMRI as the prolongation of both T1/T2 mapping times. The diffuse fibrosis observed in our case is likely explained by the efflux of collagen-1 into the extracellular space.^10^ We hypothesize that the presence of diffuse fibrosis, in the setting of hypertrophied apical segments and supportive ECG changes, tilted our diagnostic lens towards apical HCM. However, it is uncommon to observe such prolonged T1 mapping values in apical HCM as it relates to a different underlying pathophysiological process.^7,9^

**Figure 4:**
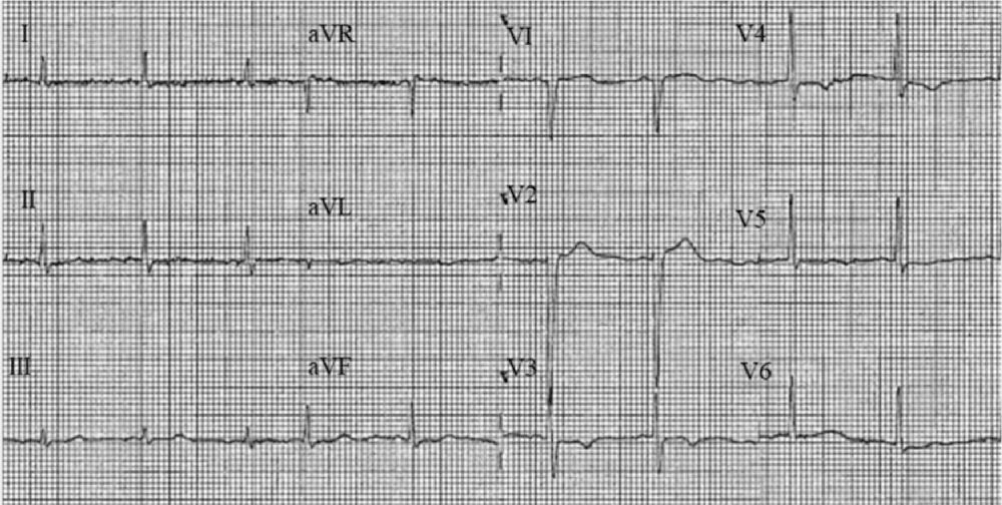
Ongoing resolution of electrocardiographic changes at 2 years.

It is interesting to observe the evolution of her ECGs, although initial concerns of an anterior non-ST elevation myocardial infarction were raised (*Figure 1F*), they evolved over several months with features in keeping with apical HCM (*Figure 2F*). These features were more pronounced at 15 months (*Figure* 3F), and continued to persist at 2 years (*Figure 4*). Cohort studies investigating acute to long term ECG changes in TCM, reported resolution of ECG changes at 12 months with a small minority persisting beyond this timeframe (see Supplementary data online, *Videos S1–S3*).^11^

## Conclusion

The phenomena of transient apical wall thickness in the recovery phase of TCM can morphologically mimic apical HCM. There can be diffuse fibrosis and ECG changes that support a diagnosis of apical HCM. Parametric cMRI mapping with T1/T2 times are crucial to differentiating TCM from apical HCM and avoids the misdiagnosis of apical wall oedema as true apical HCM.

## Lead author biography


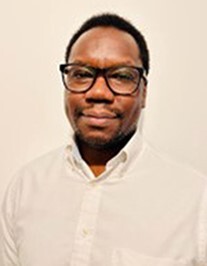


Dr Chitsa Seyani is a heart failure research fellow based at the University Hospital Southampton, Southampton, UK, specializing in heart failure and cardiac MRI.

## Supplementary Material

ytae432_Supplementary_Data

## Data Availability

The data underlying this article will be shared on reasonable request to the corresponding author.

## Supplementary material

**Consent:** Consent was sought from the legal next of kin for publication of this case report including the use of clinical images in line with COPE Guidelines.

**Funding:** No funding was used to support this work.
